# Medicine

**Published:** 1893-09

**Authors:** George Parker


### Progress of the Medical Sciences.

### MEDICINE.

The duration of incubation and infectivity in the common fevers is a matter of extreme practical importance, and the Report[1] of the Committee of the Clinical Society will be welcomed on account of the great care and skill with which it has been drawn up, and the completeness of the data on which it is based. Still there are many points in it on which further information is desirable. Whether, for instance, the *British Medical Journal*[2] is right in suggesting that the incubation of scarlatina and diphtheria may occasionally exceed seven days. The general result of the report is to confirm the ordinary belief that scarlatina, diphtheria, influenza,—and we may add, yellow fever, dengue, erysipelas, and cholera,—go through the incubation stage within one week, the normal period for the first two being 2 days, and for influenza 3 or 4 days. Those fevers which take two weeks are variola, normal period 12 days, varicella 14, measles 10, mumps 19, German measles 18, enteric 12; and we may add to these pertussis, relapsing, and typhus. Further, the evidence tends to show that the variation of maximum and minimum periods of incubation is less than is usually supposed.

In connection with these maximum instances arises the question of quarantine of healthy persons who have been exposed to infection. Dawson Williams lays down that this must be one day beyond the maximum observed period, and that the person must be at the end of it free from pyrexia and illness, and have disinfected his clothes.[3] This rule would give 8 days' quarantine in scarlatina, diphtheria, and probably influenza, 15 days in measles and variola, 20 in varicella, 24 in enteric and rubeola, and 26 in mumps. The infectivity of the diseased person cannot be fixed with equal certainty. The diphtheritic patient may convey the disease for a long and uncertain period. Scarlatina is infectious during peeling, and sometimes as long as 8 weeks; influenza for 10 days; variola until all scabs have cleared off; typhoid until 2 weeks after convalescence; mumps probably until 3 weeks from onset of parotitis; measles until 3 weeks from onset of rash; and rubeola for 14 days. Fomites may convey infection for longer periods, notably in the case of scarlatina, typhoid, and diphtheria.

1 Supp. *Trans. Clin. Soc.*, vol. xxv.

2 *Brit. Med. Journ.*, 1893, vol. i., p. 958.

3 *Med. Mag.*, June, 1893.

The medicinal treatment of malignant disease has long been such a hopeless subject that it is difficult to believe in any claims made on behalf of new remedies. Methyl blue, Chian turpentine, and Mattei fluids, with a host of others, have appeared and vanished in succession. The actual pathology is at present in the clouds; and even if the protozoa theory should be proved, we know too little of the conditions of life in these parasites to frame any plan of treatment. Moreover, the low vitality of malignant growths leads to their invasion by various organisms, particularly after ulceration has taken place. Hence apparent improvement may at times be produced by treatment affecting the new-comers, without any effect on the fatal termination. However, certain observations of Fehleisen's on undoubted cures following attacks of erysipelas have borne fruit, and Dr. Coley has tabulated 38 cases in which either improvement or absolute cure has resulted from the use of the germ of erysipelas, and others where he has used its toxic products.[1] Accounts of its efficacy date from the 17th century, as Fehleisen notices,[2] and striking results from "erysipèle salutaire" have also at times followed accidental attacks in cases of acute rheumatism, mental diseases, keloid, and particularly in lupus. Ricord attempted to treat phagedenic chancres in this manner. After Fehleisen had obtained pure cultures of the micrococcus of erysipelas, he treated seven persons suffering from sarcoma, carcinoma, and lupus by inoculation. Great diminution of the tumours, and in one or two instances a complete and permanent cure, took place. In a case of Busch's, the cells of a tumour thus destroyed were found to have undergone fatty degeneration, and to have been changed into a yellow-white emulsion.[3] In Coley's monograph, we find recorded 17 cases of carcinoma, of which 3 were said to be absolutely cured and 10 improved; also 17 cases of sarcoma, of which 7 were cured, showing no recurrence from one to seven years later, and 10 were greatly improved. In 4 other cases of either sarcoma or carcinoma, 2 cures were recorded. In all 12 cures out of 38 cases, which is of course a very fair result for a new and probably imperfect method. Some of the instances are certainly most startling and thoroughly attested. Professor Spronck, of Leyden, has carried out investigations on the same subject, and has injected 26 cases of malignant disease in parts remote from the tumour with the toxic products of the erysipelas germ. His method seems at present defective; the results were less marked, recurrence took place in almost all, but the injections were probably too small, and the activity of the fluid injured by the temperature to which it had been exposed in order to destroy the streptococci. If filtered cultures can be got to produce more permanent results, it is clear that much of the danger from using erysipelas germs themselves, and the need of

[1] *Amer. Journ. Med. Sci.*, May, 1893.

[2] *Selected Essays.* Syd. Soc., 1886.

[3] Fehleisen, *op. cit.*

isolation, will be avoided. Coley is therefore employing these cultures in the researches he is now conducting.[1]

It is scarcely necessary to remark that in spite of the care and exactness shown in these reports, and the undoubted success obtained, we have had a serious warning in our experience of Koch's method against placing too much faith in the remedy. It may be that the high temperatures produced have much to do with the changes wrought in the tumours, and we may well doubt whether a disease-process like that of erysipelas, so transient that it cannot protect against itself for more than a short period, can produce results so deep and lasting as those claimed for it. Repeated inoculations for a long period will be needed in many patients, otherwise any surviving focus may start into activity owing to the absence of a permanent immunity. The very identity of the germ in different cases of erysipelas is not completely settled. Thus Fehleisen refers to the variety of erysipelas-like diseases in rabbits, and many authorities insist that streptococcus pyogenes when injected superficially produces true erysipelas in human beings.

In addition to the thirty-eight cases where erysipelas was produced, Coley has recently been employing repeated injections of the toxic products with some success, particularly in sarcoma. He uses stronger injections than Spronck did, but does not cause a reaction lasting more than twenty-four hours. Amongst others, a sarcoma in one of his earlier patients, which had recurred, again disappeared under this treatment. Time will show whether the results are permanent.

Dr. Snow[2] advocates the continued administration of opium, which he believes to exercise "a direct and conspicuous retardative action," materially checking the cell growth if persistently given, and not merely alleviating the pain. It causes an improvement in the objective phenomena, he says, which can be obtained by no other possible method of treatment.

Another method of treatment has been put forward by Dr. J. S. Wight,[3] who recommends the administration of bromide of arsenic, not in place of operative procedures, but as an aid to them and as a safeguard against recurrence. He gives $\frac{1}{40}$ to $\frac{1}{10}$ of a grain after meals, and carbonate of lime in calumba before them for long periods, and considers that these remedies remove small foci of infection which have been left behind by the surgeon. A number of cases which he operated on from three to five years ago, and in which he gave the bromide of arsenic both before and after the operation for many months, have shown no recurrence. Other cases unfit for operation were, he thinks, benefited by its administration. He does not give any statistics, nor does he claim that the drug will remove a malignant tumour; but believing that it enables

[1] *Ther. Gaz.*, June, 1893, and Coley, *loc. cit.*

[2] *A Treatise on Cancers.* 1893. [3] *Annals of Surgery*, Ap., 1893.

the surrounding tissues to resist the spread of infection, he urges further trials of the remedy. If, however, it is capable of preventing recurrence, it is difficult to see why, if it is given early enough, it should not cure the primary infection; and with regard to his apparent cures, it may be argued that any group of cases which are taken early enough, if the growths are thoroughly excised, show a certain percentage of permanent cures. Lassar, too, reports five cases of malignant growths on the skin which appear to have been cured by the internal use of arsenic.[1] The instances are certainly remarkable, but nothing is more deceptive than the variation in growth and pain shown in the progress of many malignant tumours. Further trials may well be made of these methods; but the serious danger is that patients, hearing of apparent success by their use, may be tempted to insist on putting off operative help till the chance of recovery is lost. The *British Medical Journal* gives a salutary caution in referring to Brown-Séquard's expression of surprise that the disease which gave the greatest number of ameliorations after the use of his organic extracts was cancer superficially situated. "We confess," it remarks, "we cannot share this surprise; the recent developments of Matteism should have prepared Professor Brown-Séquard to guard against the fallacies into which he has unwittingly dropped."[2]

The success of thyroid injections in myxœdema, coupled with the discoveries of Minkowsky on the production of diabetes by the entire removal of the pancreas, have directed attention both to the pathology of diabetes and to more satisfactory methods of treatment. The probability that some cases of diabetes are due to pancreatic disease has led to the use of injections of pancreatic extract and to feeding with raw pancreas; but the results are unsatisfactory, because either the remedy has no power or the cases were not pancreatic in origin.

Pancreatic diabetes seems to be acute, running its course in from three to six weeks, occurring usually in young subjects, and may be marked by the presence of fatty stools with great emaciation and a large excretion of sugar.[3] But its diagnosis is difficult and uncertain, and many of the cases treated showed no sign whatever of a pancreatic origin. Secondly, the analogy of myxœdema gives very uncertain ground for thinking that feeding with raw pancreas would cure even pancreatic cases, and Minkowsky's experiments gave a distinct negative to the idea, while he found that the transplantation of a piece of the gland with an external sinus did do so.

Dr. Hector Mackenzie, however, held that the glycolytic functions of the pancreas may render it of some use if ad-

1 *Berl. klin. Woch.*, June 5, 1893, and *Brit. Med. Journ.*, June 17, 1893.

2 *Brit. Med. Journ.*, 1893, vol. i., p. 1279.

3 Vaughan Harley, *Brit. Med. Journ.*, 1892, vol. i., pp. 9, 1330, vol. ii., p. 452.

ministered in ordinary diabetes, even though it could not replace a diseased pancreas. This point he has tested with three patients, to whom he gave the liquor pancreaticus. He found that the lassitude, thirst, and polyuria were lessened by it.[1]

In two other cases tested by Dr. Neville Wood[2] some increase of weight took place. Dr. Hale White[3] obtained only negative results; Dr. W. K. Sibley[4] reported considerable improvement in general condition in one case from raw pancreas; but Dr. Donkin[5] alone noticed any decrease in the excretion of sugar. This was in a single case, and occurred both when raw pancreas was given and when the extract was injected. The general results in the case were unsatisfactory, and Dr. Donkin noticed that the raw pancreas may cause severe erythema, with fever and a slight sore throat. Thus little success has attended these trials, and no attempt at transplanting a pancreas has been recorded of late.

Something has, however, been done towards deciding on a perfect food for diabetics. Saundby[6] has given up the use of gluten bread on account of its expense, and still more because it often contains as much as 30 per cent. of starch. He prefers Clarke's biscuits with Callard's almond cakes for a change. Hale White[7] compares the soya bean, having only 3 per cent. starch, with gluten bread containing 16 per cent. He has employed soya beans for all his patients during the last three years. After preparation to get rid of the oil, both bread and biscuits are made from them, as well as cakes and scones. The beans themselves are used for soup. The bread must be made fresh every few days, but the biscuits keep well and are palatable. Ebstein[8] recommends aleuronat, a by-product in starch manufactories patented by Hundhausen, but cheap, and, as he thinks, far preferable to gluten bread. It contains always more than 80 per cent. of albumen and less than 7 per cent. of starch. Minkowsky also investigated the value of levulose as a food; and Leyden has shown that, while ordinary sugar is excreted unchanged by diabetics, a large part of levulose is used up in the body, and that, with moderate doses, this increases the longer it is used. Thus levulose, as found in the Jerusalem artichoke, is of great value as a food stuff; but when given in excess it is nearly as harmful as cane sugar.

It would be interesting to know whether diabetes was prevalent in this country in former times before the introduction of cane sugar as a common article of diet. Few greater changes have been made in the national dietary than this. The Jews, who consume much sugar, suffer more than others in the United States; American Indians and Chinese, we believe, very little.

[1] *Brit. Med. Journ.*, 1893, vol. i., p. 63. [2] *Ibid.*, 1893, vol. i., p. 64.

[3] *Ibid.*, 1893, vol. i., p. 452. [4] *Ibid.*, 1893, vol. i., p. 579.

[5] *Ibid.*, 1893, vol. i., p. 1265.

[6] *Birm. Med. Rev.*, May, 1893. [7] *Pract.*, May, 1893.

[8] *Deutsch. med. Woch.*, 1892, No. 19, p. 417.

The most important practical question now agitated is, How far should a strict diet be observed in gouty glycosuria? Many of these patients, as Sir Dyce Duckworth observed at Newcastle, live long and remain stout and florid. Is it reasonable to place gouty patients on a strict diet of flesh and fats?[1] On the other hand, ordinary diet floods their kidneys and blood-vessels with irritating sugar, and Ralph has maintained that to allow a little potato or fruit only injures diabetics without restoring the balance of nutrition. Yet some of the stout glycosurics break down rapidly on a strict diet. Saundby would commence with a rigid dietary, then add opium till the maximum improvement is reached, and afterwards try the effect of gradual relaxation.[2] Dr. Shingleton Smith spoke strongly on the importance of washing out the tissues of these glycosurics with water or alkaline waters, not only in order to lessen the sugar, but also to remove other poisonous waste products. This seems a truer view than that of George Harley, that saline purgatives act by sweeping the food out of the intestines so quickly as to diminish the supply of sugar, just as a restricted diet does.[3]

Indeed, the sugar is after all a secondary matter, and often disappears as the gouty condition, or the defective digestion which led to it, improves, whereas the accumulation of uric acid, oxybutyric acid, &c., on unsuitable diet may be fatal.

In the other or acute type of diabetes some patients, if a grain of sugar be given them, excrete more than a corresponding amount of sugar. These, of course, need a strict diet, otherwise the patient is starved to death by the conversion of even his proteid food into some form of sugar. Hence, too the need of seeing that these patients take sufficient food in the form of fats and albumen to make up for the outflow of sugar and the absence of starchy feeding stuffs.

GEORGE PARKER.

[1] *Deut. Med.-Zeitung*, June 5, 1893, and *Brit. Med. Journ.*, June 24, 1893.

[2] *Med. Annual*, 1893. [3] *Brit. Med. Journ.*, May 27, 1893.

